# Adults with adverse childhood experiences report greater coronavirus anxiety

**DOI:** 10.1371/journal.pone.0323401

**Published:** 2025-10-21

**Authors:** Vrinda Kalia, Katherine Knauft

**Affiliations:** 1 Department of Psychology, Miami University, Ohio, United States of America,; 2 Baylor University, Waco, Texas, United States of America; Donald and Barbara Zucker School of Medicine at Hofstra/Northwell, UNITED STATES OF AMERICA

## Abstract

**Background:**

Adults with early life adversity exhibit heightened response to threat signals in the environment, which makes them vulnerable to developing stress-related mental health problems, including anxiety disorders. Yet, the impact of the COVID-19 pandemic on adults who have experienced early life adversity is understudied. Recently, researchers have characterized dysfunctional cognitions about the pandemic, which are associated with negative mental health outcomes, as coronavirus anxiety. We conducted a study to examine the relation between exposure to early life adversity, perceived threat from COVID-19, and coronavirus anxiety.

**Methods:**

Adults (N = 975; 18–78 years of age; 585 = Women) living in the United States were recruited online in October 2020. Two forms of early life adversity, maltreatment and household dysfunction, were assessed using the Adverse Childhood Experiences scale. Participants’ state anxiety was measured using the State-Trait Anxiety Inventory, and coronavirus anxiety was measured via the Coronavirus Anxiety Scale. Three items were used to measure perceived threat from COVID-19. Additionally, as reduced flexibility is implicated in the development and maintenance of anxiety disorders, participants’ cognitive flexibility was assessed using the Cognitive Flexibility Inventory.

**Results:**

The data were analyzed using parallel mediation regression analyses. Exposure to early life adversity, in the form of maltreatment and household dysfunction, were the key predictor variables. Coronavirus anxiety and state anxiety were the outcome variables. Perceived threat from COVID-19 and cognitive flexibility were added as parallel mediators into all the regression models. The regression analyses revealed that both perceived threat from COVID-19 and cognitive flexibility mediated the relation between early life adversity and anxiety. The data demonstrate that exposure to early life adversity, in the form of maltreatment or household dysfunction, was associated with higher levels of perceived threat from COVID-19, which, in turn, predicted increased coronavirus anxiety and state anxiety. In contrast, appraisal of everyday challenges as controllable, one of the two types of cognitive flexibility assessed, predicted lower levels of coronavirus anxiety and state anxiety. However, exposure to maltreatment and household dysfunction was associated with reduced cognitive flexibility.

**Conclusion:**

This study replicates and extends prior research showing that adults with early life adversity experienced increased anxiety during the pandemic. The findings bolster existing theories that highlight the importance of threat appraisal as a mechanism for the development of anxiety disorders in this population. Additionally, this report adds to the limited body of work on the impact of COVID-19 in adults who have experienced early life adversity.

## 1. Introduction

The COVID-19 pandemic has been unprecedented. Per World Health Organization (WHO) [1] over 7 million deaths worldwide, and over 1.2 million deaths in the US, to date, have been attributed to COVID-19. The pandemic has also profoundly impacted the mental health of Americans [2]. In the course of an average adult’s life, experiences of trauma and stress are to be expected; not everyone encountering these challenges develops enduring mental health problems as a result of these stressors [3]. However, converging streams of data are beginning to show that the pandemic’s impact has been disproportionately negative on vulnerable populations and communities [4,5]. In particular, individuals in high risk groups were more likely to experience increased anxiety due to the virus during the pandemic [6].

Adults with early life adversity have been previously identified as vulnerable to stressors [7], yet have received very little attention during the COVID-19 pandemic [8,9]. Exposure to dysfunctional family interactions in early development, characterized by abuse (physical, emotional, or verbal), neglect, having parents with mental health problems (e.g. substance abuse), and family violence are broadly identified as experiences of early life adversity [10]. Early life adversity (hereafter; ELA) is implicated in developing a number of health problems later in life [11]. These include increased risk of mental illness, such as anxiety [12], as well as chronic health diseases [13]. Unfortunately, adults who have experienced ELA are not rare in the American population [14]. Per the Centers for Disease Control and Prevention (CDC), approximately 64% (or majority of the adult population) of the surveyed adults in the United States report experiencing at least 1 form of ELA [15].

### 1.1. Early life adversity and brain development

Adults who have been exposed to ELA, in the form of abuse, neglect, and household dysfunction prior to 18 years of age, have been identified as vulnerable to stressful experiences in adulthood [7,16]. One proposed mechanism underlying this vulnerability is the dysregulation of the body’s adaptive acute stress response through extreme and extended activation [7]. The extended activation of the stress response in early development results in the generation of toxic stress, which disrupts the body’s ability to maintain stability in the face of changing environmental demands (or allostasis). Consequently, structural and functional abnormalities in normative brain development, particularly the amygdala and prefrontal cortex (pFC) occur [7].

Empirical evidence demonstrates that ELA alters normative development of the amygdala [17]. Because the amygdala is essential for threat detection, it is more often activated in a home environment that is threatening to the child. Consequently, over time, the amygdala is engaged even in environments that are not stressful [18,19]. For instance, fMRI research has shown that adults who have been exposed to ELA exhibit heightened amygdala responsiveness when viewing emotional faces in comparison to adults without ELA [20]. Generally, the ability to attend to threats in the environment while attending to ongoing task demands is adaptive [21], as it protects the individual while they carry out goal-directed activities. However, exposure to maltreatment biases attentional and emotional processes toward threatening stimuli in the environment, which results in a sensitized threat system that is specialized to seek out threatening stimuli at the cost of the task at hand [22].

In addition to hyperactive amygdala, adults with ELA have an impaired pFC [7]. For instance, individuals exposed to ELA have smaller frontal brain regions than individuals without ELA [23]. The pFC experiences a protracted developmental period in humans and is flush with glucocorticoid receptors which make it sensitive to stress [7]; consequently, adults with ELA exhibit deficits in cognitive processes that are implicated in activity in the pFC [12]. For example, exposure to ELA in adults is associated with reduced cognitive flexibility, which is an executive process that allows an individual to make adaptive behavioral changes [24,25].

In our prior report, we assessed an attitudinal aspect of cognitive flexibility using a self-report measure. We observed that individuals with ELA were less likely to be flexible in their appraisal of everyday challenges as being controllable. However, for adults who had experienced maltreatment in early development, reduced flexibility in appraising everyday challenges as controllable was associated with elevated levels of state anxiety [9]. In normative development, the amygdala and the pFC would create a network that would allow the individual to evaluate environmental threats effectively [16]. This would allow adults to view controllable stressors as challenging and uncontrollable stressors as threatening. However, adults with ELA have an impaired pFC [7], and a hyperactive amygdala [16], which could enhance their propensity to be emotionally reactive to environmental threats [12]. Thus, the data in our prior report suggested that adults with ELA may be more likely to view daily life challenges as threatening and this may make them particularly vulnerable in contexts of uncontrollable stress such as a global pandemic [9].

### 1.2. Early life adversity as experiences of threat and deprivation

Understanding the impact of early adversity on health outcomes is complicated by the fact that different types of adversities (e.g., abuse, household dysfunction) tend to co-occur [10]. For example, if a child grows up in an abusive home environment they are also more likely to experience neglect from their primary caregivers. As a result, the literature has emphasized studying the cumulative effect of adverse experiences as well as different types of adversity that an individual has experienced [24]. More recently, McLaughlin [26] proposed distinguishing between experiences of deprivation (i.e. the absence of expected environmental inputs that facilitate development) and threat (i.e. the presence of experiences that represent danger) to understand the distinct ways in which different types of ELA could cumulatively impact outcomes.

Using McLaughlin’s dimensional model of deprivation and threat [26], and Dubowitz and Bennet’s [27] characterization of neglect as maltreatment we determined that reports of experiencing abuse and neglect in early development would be classified as maltreatment. Consistent with the dimensional model [28], we believe these experiences would evoke a threat response in the child with consequences for subsequent brain development. Reports of experiences with household dysfunction (e.g., separation, parental mental illness) in early development, on the other hand, would deprive the child of input needed for normative development. Within the dimensional model these experiences would be characterized as experiences of deprivation.

In our prior report [9] we studied exposure to ELA in a sample of adults (N = 356) and observed that those adults who had experienced ELA in the form of maltreatment (i.e., threat) perceived a greater threat from COVID-19 in late March 2020, when the US had 85,000 cases of COVID-19 nationwide. Further, these adults with experiences of childhood maltreatment also reported greater state anxiety. State anxiety is a temporary, and generally adaptive, increase in tension and worry in response to an adverse environmental change. Neurologically, there is evidence to suggest that state anxiety correlates with functional activity in the precuneus cortex, which is implicated in adapting behaviors to environmental changes [29]. Finally, we observed that the relationship between maltreatment and state anxiety levels was fully mediated by their perception of the threat posed by COVID-19. These effects remained significant after controlling for the effects of education, SES, age, and gender. No such relationship was observed between perceived threat from COVID-19 and state anxiety in adults who had experienced household dysfunction (i.e., deprivation) early in development. Thus, we [9] were able to show that exposure to threatening environments in early development increased reactivity under conditions of threat in adulthood.

### 1.3. Current Study

We conducted a study to replicate our previous work as reported in Kalia and colleagues [9]. Since data collection for the previous publication took place at the start of the pandemic, just as universities, colleges, schools, and businesses in the United States began to shut down, we wanted to ensure that our findings were not due to sampling error [30]. Additionally, we sought to extend the presented findings in Kalia and colleagues by examining the relation between ELA, perceived threat, and a specific type of anxiety related to the pandemic (i.e. coronavirus anxiety). In 2020, Lee developed a mental health screener that identified a coherent set of unpleasant cognitions and feelings attributed to the fear of COVID-19, named coronavirus anxiety. Coronavirus anxiety is characterized by anxiety and trauma related to the coronavirus. Individuals with coronavirus anxiety also report experiencing a wide range of psychological issues including elevated depression, generalized anxiety, hopelessness and functional impairments. Functional impairments associated with coronavirus anxiety include behaviors indicative of elevated fear and arousal such as disturbed sleep, bouts of dizziness, and loss of appetite [31]. As such, researchers have speculated that coronavirus anxiety may be an underlying risk factor for development of mental health problems during the pandemic [32] and after the pandemic is over [33].

Based on the report by Kalia and colleagues [9], our first hypothesis was that exposure to ELA, in the form of maltreatment, would predict higher levels of state and coronavirus anxiety. Additionally, past research has shown that early life stress is implicated in the development of anxiety disorders in adulthood so we predicted that ELA, in the form of household dysfunction, would predict higher levels of state and coronavirus anxiety [34]. Furthermore, ELA is implicated in having a hyperactive amygdala [16], which could mean that adults with ELA would exhibit greater sensitivity to environmental threats and respond with enhanced reactivity [12]. Thus, we predicted that the relationship between ELA and anxiety (both state anxiety and coronavirus anxiety) would be mediated by perceived threat from COVID-19. Finally, as deficits in cognitive flexibility have been observed in anxiety [35] and ELA [24,25] we also examined the role of cognitive flexibility in the relation ELA and anxiety. In our prior report we observed that reduced flexibility in appraising everyday challenges mediated the association between ELA and state anxiety. Thus, our final hypothesis was that cognitive flexibility, specifically flexibility in appraising everyday challenges as controllable, would mediate the relation between exposure to ELA (in the form of maltreatment and household dysfunction) and anxiety (both state anxiety and coronavirus anxiety).

## 2. Methods

### 2.1. Participants and procedure

All study procedures were approved by the Institutional Review Board #01620r. Because we were also interested in ensuring our findings in Kalia and colleagues [9] were generalizable we decided to recruit a much larger sample of participants using a data collection platform we had not used for the previous study [30]. Thus, 2020, between October 12^th^ and 14^th^, people living in the United States were recruited via the online platform MTurk. On MTurk potential participants were able to read a brief description of the study and then opted to participate. If the individual was interested in the study, they had to click on a brief description of the study which led them to a detailed consent form. In the consent form for this study, participants were told that researchers were interested in the role of early life experiences on their thoughts, feelings and behaviors. Participants were also informed that they would be completing several questionnaires in exchange for $2.00. At the end of the consent form participants were asked to explicitly click on ‘agree’ if they wish to proceed with the study. Clicking on ‘agree’ led the participants to the Qualtrics survey with the questionnaires. Those who were not interested in participating clicked on ‘disagree’ which took them to a thank you page that ended their engagement with the study.

At the time of data collection, per an article in CNN [36] the United States had more than 7.9 million people who had tested positive for COVID-19 with 40,000–50,000 new cases being reported per day. More than 200,000 Americans had lost their lives to the disease. The sample consisted of 975 participants (Men = 362, Women = 585, Non-binary = 1, Gender Missing = 27) after excluding 96 participants who failed more than half of the attention checks. Although the age range for the sample was wide,18–78 years, 96.8% of the participants were younger than 61 years, (*M*_*Age*_* *= 36.28, *SD *= 10.31).

A majority of the participants identified as white (61.8%), Black or African American (19.1%), Native American (6.5%), Hispanic or Latino (6.3%), Asian or Asian American (3.4%) or preferred not to disclose (3%). The vast majority of participants (95%) had at least some post-high school education, and approximately 25% of the sample held a post-undergraduate degree. To evaluate subjective socioeconomic status, we had asked participants to rank themselves from 1 (worst off with the least money, worst employment status) – 10 (best off with the most money, best employment status) on a ladder. Majority (62.3%) of the participants placed themselves at rungs 8 (19.1%), 7 (16.5%), 9 (13.4%), or 6 (13.3%). To evaluate overall health status we had used 1 item, *In general would you say your health is* (1 = excellent to 5 = poor), from the 12-item short form health survey [37]. After providing informed consent, participants completed a series of questionnaires that assessed their early life experiences, perceived threat from COVID-19, cognitive flexibility, coronavirus-related anxiety, and state anxiety before completing a demographics form.

### 2.2. Measures

#### 2.2.1. Adverse Childhood Experiences Scale (ACEs; [10]).

The ACE scale was used to measure an individual’s exposure to different categories of early life adversity experienced prior to the age of 18. Prior research by Afifi and colleagues [38] has shown the ACE scale measures two underlying factors: maltreatment and household dysfunction. To be consistent with our prior publication [9], we split the 10-item ACE scale into subscales that measured maltreatment and household dysfunction.

Five items of the ACEs scale assessed maltreatment (i.e., emotional, physical, and sexual abuse; physical and emotional neglect; *“Did a parent or other adult in the household often swear at you, insult you, put you down, or humiliate you OR act in a way that made you afraid you might be physically hurt”*) and five assessed household dysfunction (i.e., domestic violence, parental separation or divorce, substance abuse or mental illness in the home, and incarcerated family members; *“Was a household member depressed or mentally ill or did a household member attempt suicide?”*). Participants responded to each item in a binary fashion (i.e., yes or no). Each yes response was coded as a “1” and “no” responses were coded as “0”. Scores for the two subscales were summed up separately. Higher scores on each subscale represented greater exposure to maltreatment or household dysfunction in childhood.

#### 2.2.2. Cognitive Flexibility Inventory (CFI; [39]).

We used the CFI to measure self-reported cognitive flexibility. The CFI provided insight about two domains of cognitive flexibility: alternatives and control. The control subscale measured the tendency of the individual to view challenges as being within one’s control. The alternatives subscale captured the individual’s ability to come up with multiple solutions to a problem or see a problem from multiple perspectives. The CFI contains 20 items that participants responded to via a seven-point Likert scale ranging from *strongly disagree* (1) to *strongly agree* (7). Control was measured through a subscale of seven items such as *“I am capable of overcoming the difficulties in life that I face”* (α = .90). We reverse scored items as described by Dennis and Vander Wal [39] and summed responses to create a score for the Control subscale. Higher scores indicated higher levels of that facet of cognitive flexibility.

#### 2.2.3. Perceived Threat of COVID-19 [9].

Three items were drawn from prior research on pandemics [40] and have previously been used by Kalia and colleagues [9,41] to assess perceived threat. The questions were: 1) *How much have you been impacted by COVID-19?* (*1 = a little to 5 = my life has completely changed*); 2) *How serious of a problem do you think COVID-19 is?* (*1 = it’s not very serious to 5 = it is catastrophic*); and 3) *How likely is it that you would test positive for COVID-19?* (*1 = not very likely to 5 = extremely likely*). An exploratory factor analysis of the three items suggested a one factor solution (i.e., one factor had an eigenvalue >1). All three items had factor loadings exceeding.30 were retained and were retained in the composite. Ratings of the three items were summed to create an overall measure of perceived threat from COVID-19.

#### 2.2.4. State-Trait Anxiety Inventory (STAI; [42]).

To reduce participant burden, state anxiety was assessed using a 6-item version of the STAI [43]. This version of the STAI has been shown to correlate strongly with the full-length inventory (*r* = .95). Participants respond to items such as *“I feel tense”* on a 4-point Likert scale ranging (1 = *not at all* to 4 = *very much so*). Scores are summed to create an overall score, with higher scores indicating greater state anxiety (α = .74).

#### 2.2.5 Coronavirus Anxiety Scale (CAS; [31]).

Coronavirus anxiety was assessed using a five-item scale that was designed to identify COVID-related anxiety that reaches the level of dysfunction or impairment. The items asked participants about negative experiences over the last two weeks related to information or thoughts about coronavirus, such as *“I felt paralyzed or frozen when I thought about or was exposed to information about the coronavirus”*. Each item was rated on a Likert scale (0 = *not at all* to 4 = *nearly every day over the last two weeks*)*.* Scores were summed, with higher scores indicating more anxiety related to coronavirus (α = .92). According to Lee [31], scores higher than 9 on the scale were indicative of dysfunctional levels of coronavirus anxiety. Within our sample, 46.2% met or exceeded this threshold, suggesting that these individuals experienced levels of coronavirus anxiety that may be dysfunctional.

#### 2.2.6. Data analytic plan.

**Fig 1:**
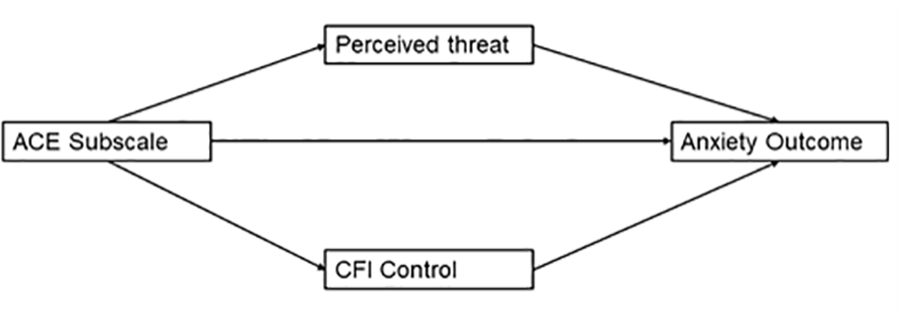
Conceptual model of proposed parallel mediation.

Parallel mediation regression models will be conducted using Hayes’ PROCESS 3 macro model 4 [44] in SPSS version 25.0. ACEs-maltreatment or ACEs-household dysfunction will serve as the independent variables in the models with state anxiety or coronavirus anxiety as the dependent variables. To be consistent with Kalia and colleagues [9], CFI-Control and perceived threat from COVID-19 will be tested as parallel mediators (See Fig 1). As CFI-Alternatives did not significantly correlate with ACEs in previous work from this line of research [see 9,24], models including CFI-Alternatives as a mediator will not be explored in the present study. Across all 4 models participant’s age, gender, subjective SES, education, overall health status, and race/ethnicity will be added as covariates. The variance inflation factors for CFI-Control and perceived threat from COVID-19 ranged from 1.089–1.535, suggesting multicollinearity between the two mediators would not be a problem within the models. A Bonferroni correction will be used to adjust for multiple comparisons. For the four models that will be presented, the adjusted α is.01250.

## 3. Results

### 3.1. Descriptive statistics and correlations

**Fig 2:**
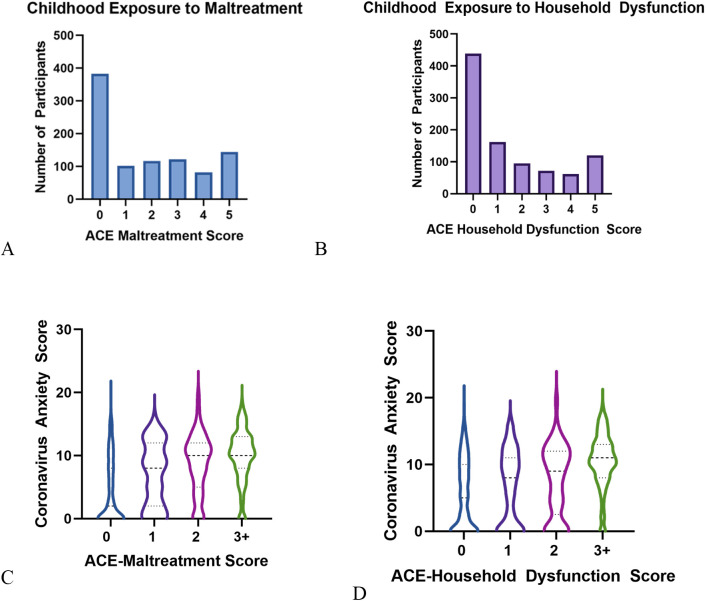
Frequencies of reported ACE-Maltreatment (A) and ACE-Household Dysfunction scores; Violin plots of associations between coronavirus anxiety and ACE-Maltreatment Scores (C) and ACE-Household Dysfunction scores (D); Within violin plots the center of each distribution is the median, which can be identified by the thicker line with larger dashes. The 25^th^ and 75^th^ quartiles can be identified by the thinner lines with smaller dashes.

Data were normally distributed (skew coefficient < |2|; kurtosis coefficient < |4|). Bivariate correlations and descriptive statistics are presented in Table 1. Consistent with prior studies [e.g., 24,9], approximately 63% of participants reported experiencing at least one form of maltreatment and 52% of participants reported experiencing at least one form of household dysfunction (See Table 2 and Fig 2).

**Table 1:** Correlations and descriptive statistics.

	1.	2.	3.	4.	5.	6.	7.	8.	9.	10.	11.
1. ACE-Maltreatment	–										
2. ACE-Household Dysfunction	.81***	–									
3. Perceived Threat	.14***	.10**	–								
4. CFI Control	−.41***	−.32***	−.27***	–							
5. CFI Alternatives	.06	.08*	.18***	.001	–						
6. Coronavirus Anxiety	.47***	.39***	.42***	−.70***	.06*	–					
7. State Anxiety	.37***	.36***	.21***	−.56***	−.17***	.49***	–				
8. Age	−.03	.01	−.01	.12***	.06	−.10**	−.06	–			
9. SES	.29***	.19***	.15***	−.45***	.03	.51***	.17***	−.04	–		
10. Education	.11**	.02	.15***	−.29***	.04	.30***	.12***	−.08*	.41***	–	
11. General Health	−.14***	−.15***	.05	.05	.18***	.02	−.27***	−.01	.21***	.15***	–
Mean	1.84	1.49	8.42	26.25	71.87	7.25	12.39	36.28	6.99	3.97	3.72
SD	1.88	1.80	2.29	9.22	9.39	5.31	3.54	10.31	2.05	1.01	0.86
Skew	0.49	.90	0.06	0.92	−0.52	−0.04	−0.11	1.06	−0.46	−1.22	−0.19
Kurtosis	−1.25	−0.66	−0.42	−0.10	0.55	−1.23	−0.57	−.62	−0.36	2.43	−0.54

*Note.* *p < .05 **p < .01; ***p < .001; Education: 1 = Some high school, 8 = Doctorate (PhD, MD, etc); The final dataset had 944 participants with information on state anxiety and 937 participants with information on coronavirus anxiety.

**Table 2:** Frequencies of adverse childhood experiences.

	Frequency (%)		
	Women (n = 362)	Men (n = 585)	Total (N = 975)
**Adverse Childhood Experiences**			
Emotional abuse	152 (42.0%)	262 (44.8%)	415 (42.6%)
Physical abuse	116 (32.0%)	214 (36.6%)	330 (33.8%)
Sexual abuse	111 (30.7%)	183 (31.1%)	293 (30.1%)
Emotional neglect	122 (33.7%)	223 (38.1%)	345 (35.4%)
Physical neglect	135 (37.3%)	236 (40.3%)	372 (38.2%)
Parental separation or divorce	114 (31.5%)	176 (30.1%)	290 (29.7%)
Domestic violence against parent	95 (26.2%)	189 (32.3%)	284 (29.1%)
Household alcohol/drug abuse	113 (31.2%)	209 (35.7%)	322 (33.0%)
Mental illness in household	99 (27.3%)	178 (30.4%)	277 (28.4%)
Incarcerated family member	79 (21.8%)	164 (28.4%)	243 (24.9%)
**Adverse Childhood Experiences Score**			
0	119 (32.9%)	190 (32.5%)	312 (32.0%)
1	50 (13.8%)	57 (9.7%)	108 (11.1%)
2	28 (7.7%)	55 (9.4%)	83 (8.5%)
≥ 3	164 (45.3%)	282 (48.3%)	446 (45.7%)

*Note.* 28 participants were non-binary or did not disclose their gender.

### 3.2. Parallel mediation models with maltreatment as the predictor variable

In both models, maltreatment and the covariates accounted for 5% of the variance in perceived threat, *F*(7, 936) = 6.41, *p* < .001. Maltreatment, *B* = 0.14, *t*(936) = 3.40, *p* < .001, and educa*t*ion, *B* = 0.24, *t*(936) = 2.95, *p* = .003, emerged as significant predic*t*ors of increased threat perception. Maltreatment and the covariates accounted for 32% of the variance in CFI-Control, *F*(7, 936) = 63.08, *p* < .001. Maltreatment, *B* = −1.40, *t*(936) = −9.83, *p < *.001, age, *B* = 0.08, *t*(936) = 3.15, *p* = .002, education, *B* = −1.26, *t*(936) = −4.60, *p* < .001, SES, *B* = −1.46, *t*(936) = −10.26, *p* < .001, and general heal*t*h, *B* = 0.98, *t*(936) = 3.29, *p* = .001, emerged as significan*t* predictors of CFI-Con*t*rol. Increased exposure to maltrea*t*ment, higher education and subjective SES predicted lower scores on CFI-Control, whereas older and healthier participants had higher scores on CFI-Control.

#### 3.2.1. Maltreatment predicts state anxiety.

**Fig 3:**
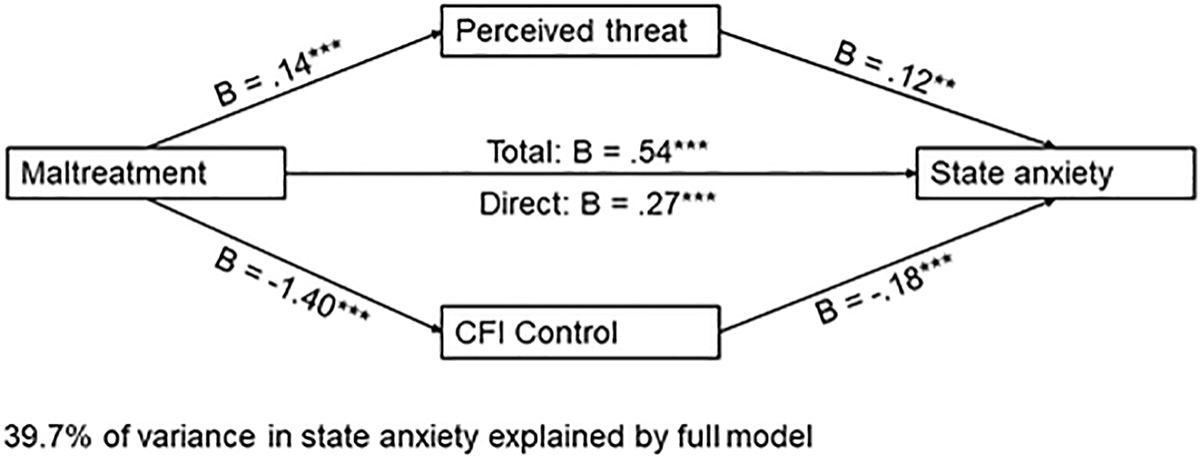
Parallel mediation model with ACE-Maltreatment as the independent variable and state anxiety as the dependent variable. *Note.* Figure represents data from n = 944 participants; *p < .01250 **p < .01; ***p < .001.

Maltreatment, the two mediators, and the covariates accounted for 40% of the variance in state anxiety, *F*(9, 934) = 68.28, *p* < .001. Both perceived threat, *B* = 0.12, *t*(934) = 2.97, *p* = .003, and CFI-Con*t*rol, *B* = −.18, *t*(934) = −15.41, *p* < .001, emerged as significan*t* mediators. Higher perceived threat was associated with increased state anxiety, whereas higher scores on CFI-Control were associated with lower state anxiety. The total effect of maltreatment on state anxiety (excluding mediators) was significant, *B* = 0.54, *t*(936) = 9.32, *p* < .001. When mediators were included in *t*he model, the direct effect of maltreatment on state anxiety remained significant, *B* = 0.27, *t*(934) = 4.96, *p* < .001. A 95% bias-corrected confidence interval based on 10,000 boo*t*strapped samples indicated that the indirect effect through threat perception, **B* *= .02, *SE* = .03, 95% CI: [.004,.036] was above zero. Likewise, the indirect through CFI-Control, *B* = .26, *SE* = .03, 95% CI: [.20,.32] was above zero. Thus, both indirect effects were significant (See Fig 3).

#### 3.2.2. Maltreatment predicts coronavirus anxiety.

**Fig 4:**
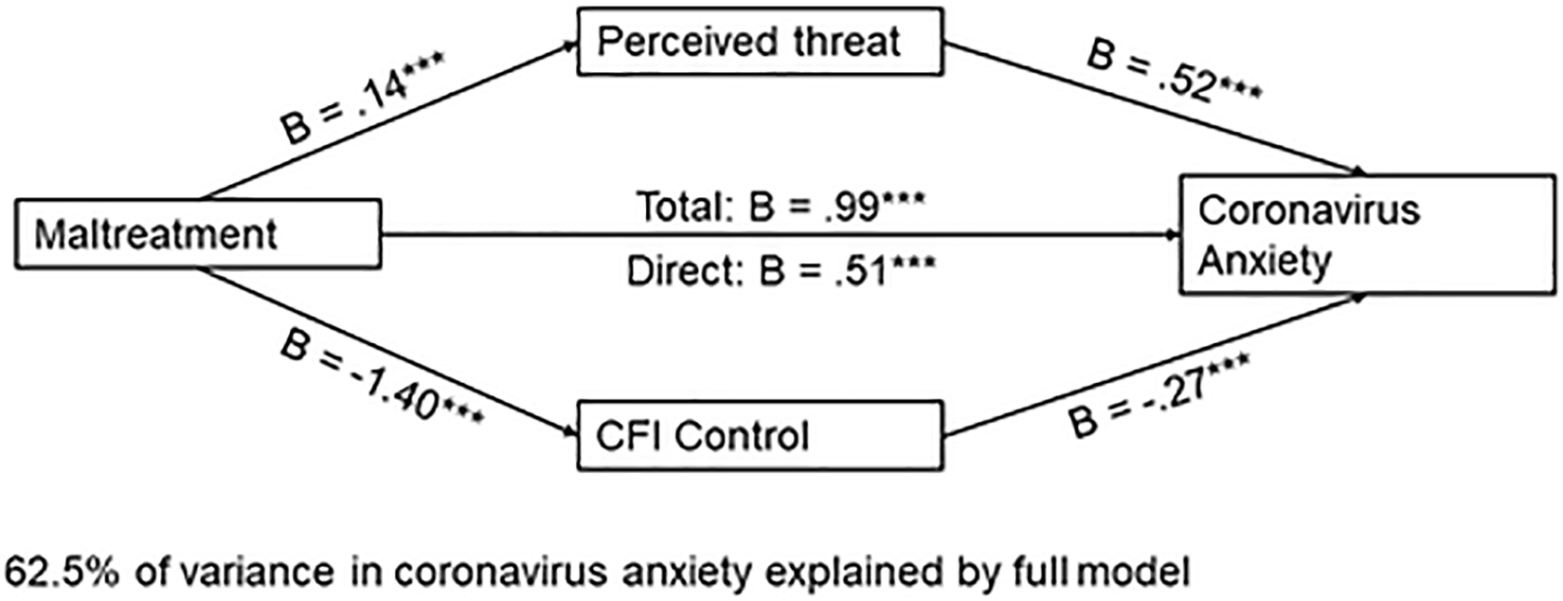
Parallel mediation model with ACE-Maltreatment as the independent variable and coronavirus anxiety as the dependent variable. *Note.* Figure represents data from n = 937 participants; *p < .01250 **p < .01; ***p < .001.

Maltreatment, the mediators, and the covariates accounted for 62% of the variance in coronavirus anxiety, *F*(9, 927) = 171.42, *p* < .001. Both perceived threat, *B* = 0.52, *t*(927) = 10.65, *p* < .001, and CFI-Con*t*rol, *B* = −.27, *t*(927) = −18.76, *p* < .001, emerged as significant media*t*ors. Higher perceived threat was associated with increased coronavirus anxiety, whereas elevated CFI-Control was associated with lower scores on the coronavirus anxiety scale. The total effect of maltreatment on coronavirus anxiety (excluding mediators) was significant, *B* = 0.99, *t*(929) = 12.74, *p* < .001. When mediators were included in the model, *t*he direct effect of maltreatment on coronavirus anxiety remained significant, *B* = 0.53, *t*(927) = 8.27, *p* < .001. A 95% bias-corrected confidence interval based on 10,000 boots*t*rapped samples indicated that the indirect effect through threat perception, **B* *= .08, *SE* = .02, 95% CI: [.033,.127] was above zero. Likewise, the indirect through CFI-Control, *B* = .38, *SE* = .04, 95% CI: [.30,.466] was above zero. Thus, both indirect effects were significant (See Fig 4).

### 3.3. Parallel mediation models with household dysfunction as the predictor variable

In both models, household dysfunction and the covariates accounted for 4% of the variance in perceived threat, *F*(7, 936) = 5.66, *p* < .001. Household dysfunction, *B* = 0.11, *t*(936) = 2.57, *p* = .010, and educa*t*ion, *B* = 0.25, *t*(936) = 3.08, *p* = .002, emerged as significan*t* predictors of increased threat perception. Household dysfunction and the covariates accounted for 30% of the variance in CFI-Control, *F*(7, 936) = 56.89, *p* < .001. Household dysfunction, *B* = −1.17, *t*(936) = −7.98, *p < *.001, age, *B* = 0.08, *t*(936) = 3.32, *p* < .001, educa*t*ion, *B* = −1.36, *t*(936) = −4.87, *p* < .001, subjec*t*ive SES, *B* = −1.62, *t*(936) = −11.48, *p* < .001, and general health, *B* = 1.13, *t*(936) = 3.77, *p* < .001, emerged as significant predictors of CFI-Con*t*rol. More household dysfunction, higher education and subjec*t*ive SES predicted lower scores on CFI-Control, whereas older and healthier participants had higher scores on CFI-Control.

#### 3.3.1. Household dysfunction predicts state anxiety.

**Fig 5:**
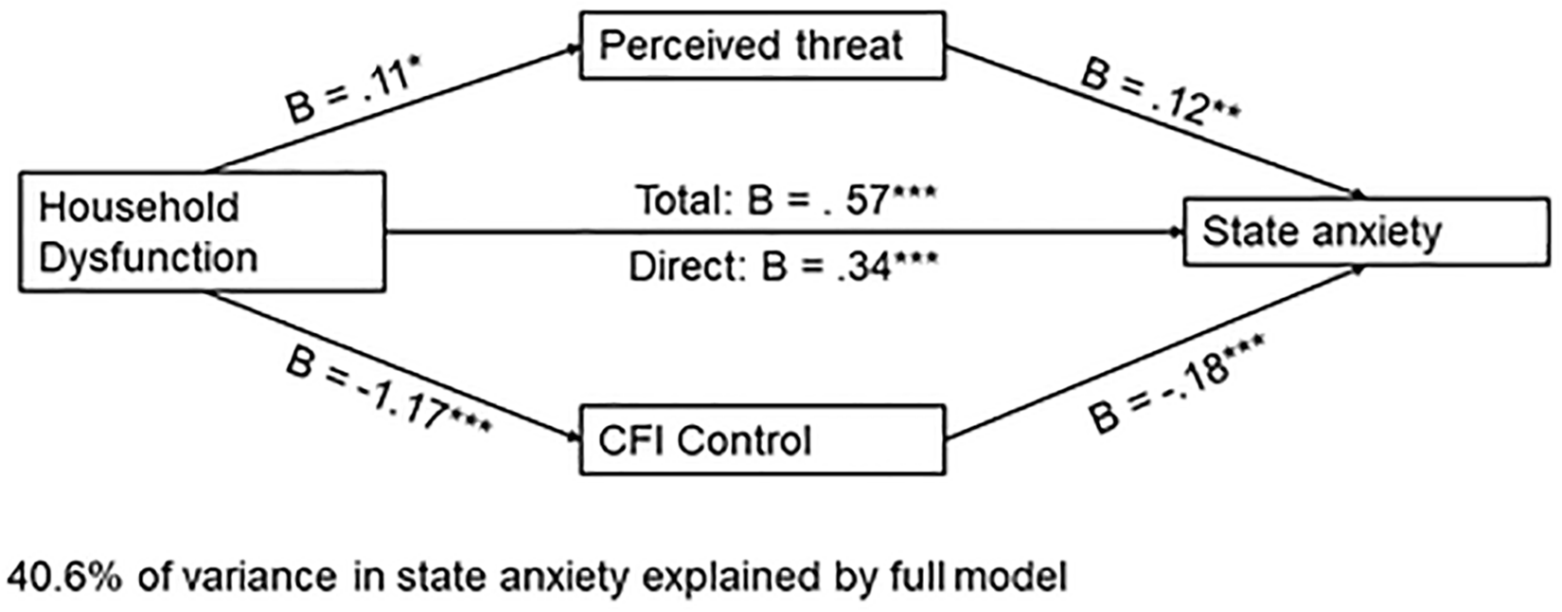
Parallel mediation model with ACE-Household Dysfunction as the independent variable and state anxiety as the dependent variable. *Note.* Figure represents data from n = 944 participants; *p < .01250 **p < .01; ***p < .001.

Household dysfunction, the mediators, and the covariates accounted for 41% of the variance in state anxiety, *F*(9, 934) = 71.06, *p* < .001. Both perceived threat, *B* = 0.12, *t*(934) = 3.01, *p* = .003, and CFI-Con*t*rol, *B* = −.18, *t*(934) = −15.72, *p* < .001, emerged as significan*t* mediators. Higher perceived threat was associated with increased state anxiety, whereas higher scores on CFI-Control were associated with lower state anxiety. The total effect of household dysfunction on state anxiety (excluding mediators) was significant, *B* = 0.57, *t*(936) = 9.61, *p* < .001. When mediators were included in *t*he model, the direct effect of household dysfunction on state anxiety remained significant, *B* = 0.34, *t*(934) = 6.32, *p* < .001. A 95% bias-corrected confidence interval based on 10,000 boo*t*strapped samples indicated that the indirect effect through threat perception, **B* *= .01, *SE* = .01, 95% CI: [.002,.031] was above zero. Likewise, the indirect through CFI-Control, *B* = .22, *SE* = .03, 95% CI: [.17,.27] was above zero. Thus, both indirect effects were significant (See Fig 5).

#### 3.3.2. Household dysfunction predicts coronavirus anxiety.

**Fig 6:**
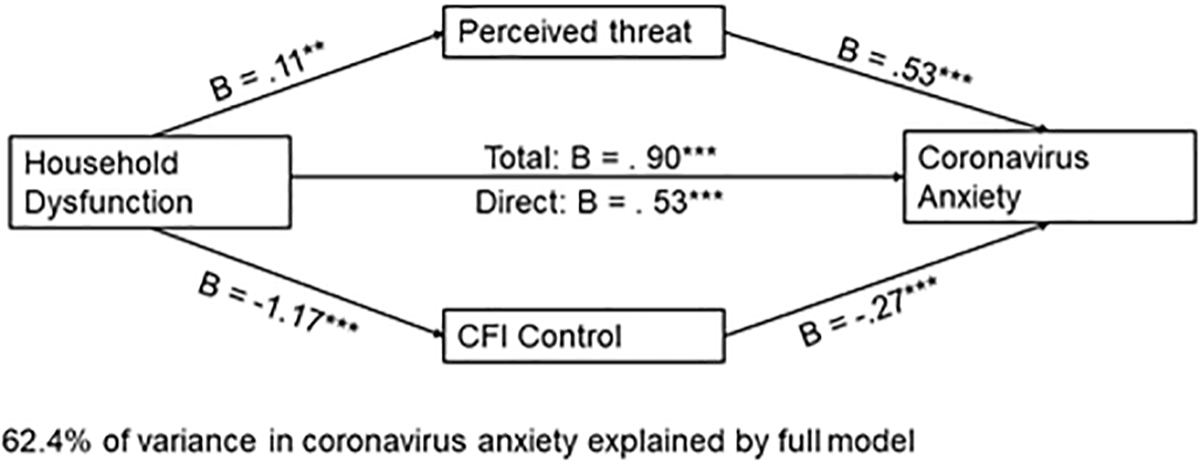
Parallel mediation model with ACE-Household Dysfunction as the independent variable and coronavirus anxiety as the dependent variable. *Note.* Figure represents data from n = 937 participants; *p < .01250 **p < .01; ***p < .001.

Household dysfunction, the mediators, and the covariates accounted for 62% of the variance in coronavirus anxiety, *F*(9, 927) = 171.08, *p* < .001. Both perceived threat, *B* = 0.53, *t*(927) = 10.82, *p* < .001, and CFI-Con*t*rol, *B* = −.27, *t*(927) = −19.46, *p* < .001, emerged as significant. Higher perceived *t*hreat was associated with more coronavirus anxiety, while elevated CFI-Control was associated with lower coronavirus anxiety. The total effect of household dysfunction on coronavirus anxiety was significant, *B* = 0.91, *t*(929) = 11.41, *p* < .001. When mediators were included in the model, *t*he direct effect of household dysfunction on coronavirus anxiety remained significant, *B* = 0.52, *t*(927) = 8.20, *p* < .001. A 95% bias-corrected confidence interval based on 10,000 boots*t*rapped samples indicated that the indirect effect through threat perception, **B* *= .06, *SE* = .02, 95% CI: [.016,.110] was above zero. Likewise, the indirect through CFI-Control, *B* = .32, *SE* = .04, 95% CI: [.25,.40] was above zero. Thus, both indirect effects were significant (See Fig 6).

## 4. Discussion

The results of the mediation regression analyses demonstrated that maltreatment and household dysfunction predicted increased state anxiety and coronavirus anxiety via greater perceived threat of COVID-19 and reduced cognitive flexibility. These data replicate our work in Kalia and colleagues [9] showing that enhanced perceived threat and reduced cognitive flexibility mediated the relationship between ACE-maltreatment and state anxiety. Our findings are also consistent with other reports showing that adults with ELA have experienced psychological distress during the COVID-19 global pandemic [45,46].

Intriguingly, the presented data show that both ACE-maltreatment and ACE-household dysfunction predict increased state anxiety whereas Kalia and colleagues [9] only observed a significant association between ACE-maltreatment and state anxiety. This difference may be due to the timing of data collection. Data collection for our first study reported in Kalia and colleagues [9] was in March 2020 when the US population was split in their appraisal of the threat posed by the pandemic [47]. Since adults with exposure to early life maltreatment exhibit enhanced neural response to environmental threats even when they are ambiguous [48], it is plausible that the anxiety that maltreated adults reported in the early part of the pandemic is due to a vulnerability conferred specifically because of the impact of maltreatment on the development of the amygdala-prefrontal circuitry [18,16].

However, the pandemic had dramatically expanded and intensified by October 2020 when we collected data for this study. Thus, it is possible that the observed association between ELA and anxiety is a consequence of the sustained activation of the stress systems leading to increased allostatic load [7]. One interesting proposal is that long periods of intense stress disrupt the excitation-inhibition balance of the amygdala that was already vulnerable because of exposure to early life stress [49]. Since we did not conduct a neuroimaging study we cannot address this issue directly. However, the fact that enhanced activity in the amygdala is associated with anxiety [50], and perceived threat partially mediated the relation between ELA and anxiety in our work should provide impetus for human neuroscience research to systematically study the impact of the pandemic on the amygdala-prefrontal circuitry in adults with ELA.

As we did not collect data on trait levels of anxiety, we cannot make any claims about an individual’s propensity to experience anxiety [51]; but, we found that both maltreatment and household dysfunction are associated with increased state anxiety and coronavirus anxiety during the pandemic. This may suggest that studying subtypes of adversity is insightful; but perhaps less informative about differential relations with outcomes in the midst of an extremely stressful chronic life event (e.g., global pandemic). Thus, our work provides some support for the notion that both experiences of threat and deprivation present themselves as questions of survival [52], such that in adults who have experienced ELA it may increase the probability of enhanced reactivity during stressful experiences.

We also examined coronavirus anxiety in relation to ELA. The coronavirus anxiety scale (CAS) captures dysfunctional anxiety associated with COVID-19, and high scores on the CAS are associated with functional impairment and distress [31,32]. The relation between ELA and coronavirus anxiety was partially mediated by perceived threat of COVID-19 [9,41]. Adults with ELA were more likely to report greater threat from COVID-19 which partially accounted for their higher scores on the coronavirus anxiety scale. Since the amygdala is implicated in threat processing [53], our data are concordant with neuroimaging research showing that increased sensitivity of the amygdala to threat may mediate anxiety in adults with ELA [50]. To the best of our knowledge, we are the first to observe that adults with ELA also experienced increased levels of coronavirus anxiety. Considering that adults with ELA are vulnerable to developing anxiety disorders [50] and cognitions about COVID-19 may predict persistent negative mental health outcomes past the pandemic [33], our work suggests the need for further research on the relation between ELA and coronavirus anxiety. Despite being adequately powered, this novel finding must be replicated before any firm claims can be made about the relationship between ELA and coronavirus anxiety.

Consistent with Kalia and colleagues [9] we observed that exposure to ELA was associated with reduced cognitive flexibility. Additionally, cognitive flexibility partially mediated the association between ELA and anxiety, such that lower levels of flexibility predicted higher levels of anxiety. Our results provide further support for the notion that deficits in cognitive flexibility are associated with anxiety [35]. Because cognitive flexibility can be enhanced via exposure to enriching environmental experiences [54], future research should examine whether increasing cognitive flexibility can aid with reducing anxiety in adults with ELA.

Since scores on CFI-Control are an indicator of an individual’s ability to appraise everyday challenges as controllable [39], our findings indicate that adults with ELA are more likely to view future challenges as uncontrollable, which increases their anxiety. This is concordant with the proposal that experiencing a lack of control over future events (i.e., everyday challenges) precludes an individual from feeling that they have the resources to avert an aversive event, which can enhance anxiety [55]. Our data were collected in the midst of a global pandemic, so it is relevant to point out that feeling some anxiety during stressful events can be adaptive as it appropriately allows attention to focus on the threat in the environment [53]. However, as seen in Fig 2, adults with 2 or more ACE-maltreatments and 3 or more ACE-household dysfunction had maladaptive levels (i.e., scores higher than 9 per [31]) of coronavirus anxiety.

Overall, our results suggest that adults with ELA are vulnerable to anxiety during stressful circumstances because they experience a greater degree of threat from an environmental stressor and are less likely to feel in control during the stressful event.

### 4.1. Limitations and conclusions

There are several limitations that must be acknowledged when interpreting our findings. First, our sample size is large but not representative of the population of the United States (e.g., disproportionately female and younger). Additionally, our data collection was restricted to individuals living in the United States and the findings may not generalize to other countries. Second, it is important to point out that the ACE scale measures early life adversity retrospectively which makes it susceptible to errors of recall. Prospective longitudinal studies should be conducted to fully understand the relationship between early life adversity and coronavirus anxiety. Third, we used a self-report measure of cognitive flexibility so our results may not extend to behavioral measures of cognitive flexibility. Though all the measures we used are either well-established (e.g., state anxiety) or have been used for replication (i.e., perceived threat), our data are correlational in nature and emerged from self-report measures; thus no causal claims can be made. Finally, it is important to note that we were unable to account for many factors that may also have contributed to a person’s coronavirus anxiety in the present study. Although our measure of perceived threat of COVID-19 does encompass a person’s feelings of how much they have personally been impacted by the pandemic, future examinations of how specific COVID-related experiences, such as the loss of close others during the pandemic, are warranted. Nevertheless, our work demonstrates that adults with ELA are vulnerable to debilitating anxiety during the pandemic and should be carefully considered when the psychological toll of COVID-19 is evaluated.
