# Supplementary material for: Adults with adverse childhood experiences report greater coronavirus anxiety
Source: PLoS One. 2025 Oct 21;20(10):e0323401. doi: 10.1371/journal.pone.0323401 (PMC12539740; doi:10.1371/journal.pone.0323401)
Supplement: Attachment — Submitted filename: ELA-CAS-Rebuttal-Response-to-Reviews.docx [file pone.0323401.s001.docx]

Dear Joven Serdoncillo,

Thank you for your email. I apologize for any miscommunication from my end. I went through my records and found the response letter and the updated manuscript we had submitted to Plos One in the last round. Below you will find the letter we had written in response to the reviews.

In this new version of the manuscript that I submitted in 2025, the following have been updated:

1. First paragraph of the introduction including an updated literature review to reflect the current status of the literature.
2. Updated references that reflect the current status of the literature.

The rest of the manuscript remains unchanged. Please let me know if you need additional information from me or have any questions. I would be happy to provide any further information to move this manuscript forward.

All the best,

Vrinda Kalia, Ph.D.

Associate Professor, Department of Psychology

Miami University, Oxford, Ohio

Dear Dr. Sar,

We thank you the reviewer for the opportunity to revise the manuscript. We appreciated the feedback we got and have made the changes recommended by the reviewer. Below we list each comment by the reviewer in order and our response is presented in ***bolded italics.***

Along with this letter we have uploaded a manuscript draft that includes all the changes made in response to reviewer feedback in blue ink so they are easier to identify. Please let me know if you need additional information from me.

1. The manuscript is written scientifically. The paper incorporates details required in terms of research methods, design, hypothesis, data collection process, analysis, results, discussions and limitations of the study. The findings of this research support previously studied outcomes. The paper is relevant for the researchers, practitioners, and students to understand the early life factors while addressing the anxiety disorders induced by uncertain situations such as COVID 19 pandemic. It would be great to incorporate possible interventions and additional research findings to address the burden of anxiety to the persons facing the pandemic with early life adversities so that practitioners can look into such aspects in their therapy processes. The paper is recommended for publication with minor revision.

***We appreciate this feedback from the reviewer and hope that clinicians and practitioners find our paper useful. We have incorporated some specific language about interventions (see pgs. 21, 22, 23).***

2. Was there any process to measure the response rate? – it would be nice to mention the response rate of the survey.

***We thank the reviewer for this question. Because of the recruitment platform used, we do not have access to the response rate data. The Amazon Mechanical Turk (MTurk) platform allows participants to browse available tasks and studies and opt in to participation and does not provide researchers with the numbers or information about participants who did not respond after seeing our study advertisement. Of individuals who began the survey, there was a 5% dropout rate.***

3. What about the missing data? How many? How the missing information/data was treated?

***We thank the reviewer for this question. As part of the consent process, participants were told they can skip questions in the survey without penalty. Of the 975 participants, 27 were elected not to provide demographic data (gender, age, education, SES, General Health). As this constituted less than 5% of the sample and the covariates have been found to be related to our outcomes of interest, these participants were excluded from analyses that included covariates (i.e., mediation analyses). An additional 2 participants were missing coronavirus anxiety scale (CAS) scores and were excluded from the mediation analyses wherein the CAS was the dependent variable.***

4. What might be the reasons for the low response rate of elderly people (above 61 years)? How it could have been improved? – it would be nice to mention in the limitation section.]

***We thank the reviewer for this important question. We suspect that one reason for the low response rate of those 61 years and older is the demographics of the Amazon Mechanical Turk (MTurk) platform, which was used to recruit participants. Though there is increasing work using MTurk samples that focus on midlife and older adults (Ogletree & Katz, 2021), comparisons between MTurk samples and population-based samples (e.g., National Election Studies samples) have found that the average age of individuals on MTurk is significantly lower than the average age of those in population-based samples (Levay et al., 2016). In a systematic review of online recruitment of older adults, Remillard and colleagues (2014) found that use of paper fliers and recruitment via email are promising alternatives for targeting older adult samples. Thus, more direct recruitment of older adults via email, fliers, or targeted advertisements could be used in the future to increase the response rate of older adults. However, the authors do note that limited internet access continues to be a problem for online recruitment of older adults and that effectiveness of online recruitment of older adults appears to vary by region. A line noting the low number of participants over the age of 61 and suggesting future directions has been added to the limitations section. See page 23.***

5. Could you please explain about sampling process? Which method or formula was used to define the sample size? Recruitment process of participants? Were there any inclusion and exclusion criteria of the participants?

***We thank the reviewer for these questions. Participants were recruited on MTurk (details provided in Methods section page 8). Participants who were English speaking, 18 years or older, and living in the United States were recruited. No other inclusion criteria were used. All participants who met the inclusion criteria and completed the surveys were included in the final sample. No specific exclusion criteria were used, only data from participants who failed more than half the attention checks was not included in the presented data. See page 8.***

6. Which geographic areas were chosen? How do you support the generalizability of the study findings to overall US population? Is this a representative sample for the entire US population and socio-economic-cultural diversities?

***Beyond limiting participation to only participants in the United States, we did not place any constraints on the geographical regions from which individuals could participate. We also did not ask which geographical regions participants were from. Thus, the sample cannot be considered a representative sample for the entire US population. A line stating this limitation has been added to the Discussion section. See page 23.***

7. It would be nice to put an exact value than an approximate one or delete the sentence if not exactly monitored – approximately 25% of the sample held a post-undergraduate degree (line – 159).

***We thank the reviewer for this suggestion. We have added exact values instead of or in addition to approximate percentages in this instance and throughout the Methods section. See page 8.***

8. What was the finding of the overall health status of the studied population (see lines 163-165)? It seems the outcomes has been missed to present.

***We thank the reviewer for this question. The data on overall health status within the studied population has been added to the participant section following the discussion of the measure. See page 9. The overall health variable was primarily included as a control variable in the regression analyses as it correlates significantly with ACE-Maltreatment, ACE-Household Dysfunction, and state anxiety (See Table 1).***

9. Researchers asked the questions related to childhood trauma (sexual abuse, torture, domestic violence, emotional abuse etc.). Opening of the unhealed wounds since childhood harms the mental health of the participants. Despite the consent and ethical clearances, the researchers have to place a duty of care and apply do no harm approach in any mental health-related research. Please explain the process of support or referrals to the participants requiring mental health services after your survey. Or, explain in the limitations, if any provisions were not put in place.

***We thank the reviewer for raising this important concern. We agree that it is crucial to extend care and appropriate resources towards participants when asking questions related to childhood trauma. In order to minimize any potential harm to participants, we take a two-fold approach. First, participants are told in the consent form that questions throughout the study reference early childhood adversity and may be emotionally evocative or disturbing, and participants are then told that participants can skip questions that they find troubling. This first step allows participants who feel that being exposed to these questions may be harmful to opt out of the survey or emotionally prepare themselves if they choose to continue. Second, in our debriefing form we conclude by acknowledging that some of the questions asked were emotionally evocative. We then urge participants to reach out to someone if they are feeling upset or anxious following the study. We provide several avenues for participants to follow should they desire mental health services, including through their healthcare provider and several free, 24 hour helplines that participants may access. A line has been added explaining this consent and debriefing process within the Methods section. See page 9.***

10. Data analysis process and presentation of findings are adequate and representative as per the design of the research.

***We thank the reviewer for this comment.***

11. Put exact number or % rather than an approximate value - approximately 63% of participants reported experiencing at least one form of maltreatment (line – 246).

***We thank the reviewer for this recommendation. Exact numbers have been added to the results section to supplement the percentages. See page 13.***

12. Explain the possible reasons and explanations for the result of ‘older participants had higher scores on CFI-Control, and educated people had lower CFI-Control’ (line 272-273), if any casual factors were studied by other researchers. Otherwise, it would be nice to mention that further studies on these aspects are required.

***We thank the reviewer for this comment. Both the correlations replicate prior work Kalia & Knauft (2020) and have now been discussed on pages 22, 23.***

13. Always mention the full form of an abbreviated text such as fMRI, US, COVID-19 etc.

***We thank the reviewer for this suggestion, the manuscript has been checked and all abbreviated text has also been presented in the full form when first mentioned.***
