# Supplementary material for: Adults with adverse childhood experiences report greater coronavirus anxiety
Source: PLoS One. 2025 Oct 21;20(10):e0323401. doi: 10.1371/journal.pone.0323401 (PMC12539740; doi:10.1371/journal.pone.0323401)
Supplement: Peer review history

# Author comment: Author response to Decision Letter 0

**DOI:** 10.1371/journal.pone.0323401.r001

15 May 2025

Attachment Submitted filename: ELA-CAS-Rebuttal-Response-to-Reviews.docx

# Peer review: Decision Letter 0

**DOI:** 10.1371/journal.pone.0323401.r002

**Authors:** Rosemary Bassey

24 Jun 2025

PONE-D-25-13890 Adults with Adverse Childhood Experiences Report Greater Coronavirus Anxiety PLOS ONE

Dear Dr. Kalia,

Thank you for submitting your manuscript to PLOS ONE. After careful consideration, we feel that it has merit but does not fully meet PLOS ONE’s publication criteria as it currently stands. Therefore, we invite you to submit a revised version of the manuscript that addresses the points raised during the review process.

==============================

**ACADEMIC EDITOR: Please insert comments here and delete this placeholder text when finished.** Be sure to: Address all the reviewer's comments in your revised submission.​

==============================

Please submit your revised manuscript by Aug 08 2025 11:59PM. If you will need more time than this to complete your revisions, please reply to this message or contact the journal office at plosone@plos.org. When you're ready to submit your revision, log on to https://www.editorialmanager.com/pone/ and select the 'Submissions Needing Revision' folder to locate your manuscript file.

Please include the following items when submitting your revised manuscript:

- A rebuttal letter that responds to each point raised by the academic editor and reviewer(s). You should upload this letter as a separate file labeled 'Response to Reviewers'.
- A marked-up copy of your manuscript that highlights changes made to the original version. You should upload this as a separate file labeled 'Revised Manuscript with Track Changes'.
- An unmarked version of your revised paper without tracked changes. You should upload this as a separate file labeled 'Manuscript'.

If you would like to make changes to your financial disclosure, please include your updated statement in your cover letter. Guidelines for resubmitting your figure files are available below the reviewer comments at the end of this letter.

If applicable, we recommend that you deposit your laboratory protocols in protocols.io to enhance the reproducibility of your results. Protocols.io assigns your protocol its own identifier (DOI) so that it can be cited independently in the future. For instructions see: https://journals.plos.org/plosone/s/submission-guidelines#loc-laboratory-protocols. Additionally, PLOS ONE offers an option for publishing peer-reviewed Lab Protocol articles, which describe protocols hosted on protocols.io. Read more information on sharing protocols at https://plos.org/protocols?utm_medium=editorial-email&utm_source=authorletters&utm_campaign=protocols.

We look forward to receiving your revised manuscript.

Kind regards,

Rosemary Bassey, Ph.D.

Academic Editor

PLOS ONE

Journal Requirements:

When submitting your revision, we need you to address these additional requirements.

1. Please ensure that your manuscript meets PLOS ONE's style requirements, including those for file naming. The PLOS ONE style templates can be found at

https://journals.plos.org/plosone/s/file?id=wjVg/PLOSOne_formatting_sample_main_body.pdf and

https://journals.plos.org/plosone/s/file?id=ba62/PLOSOne_formatting_sample_title_authors_affiliations.pdf.

2. We note that you have indicated that there are restrictions to data sharing for this study. For studies involving human research participant data or other sensitive data, we encourage authors to share de-identified or anonymized data. However, when data cannot be publicly shared for ethical reasons, we allow authors to make their data sets available upon request. For information on unacceptable data access restrictions, please see http://journals.plos.org/plosone/s/data-availability#loc-unacceptable-data-access-restrictions.

Before we proceed with your manuscript, please address the following prompts:

a) If there are ethical or legal restrictions on sharing a de-identified data set, please explain them in detail (e.g., data contain potentially identifying or sensitive patient information, data are owned by a third-party organization, etc.) and who has imposed them (e.g., a Research Ethics Committee or Institutional Review Board, etc.). Please also provide contact information for a data access committee, ethics committee, or other institutional body to which data requests may be sent.

b) If there are no restrictions, please upload the minimal anonymized data set necessary to replicate your study findings to a stable, public repository and provide us with the relevant URLs, DOIs, or accession numbers. Please see http://www.bmj.com/content/340/bmj.c181.long for guidelines on how to de-identify and prepare clinical data for publication. For a list of recommended repositories, please see https://journals.plos.org/plosone/s/recommended-repositories. You also have the option of uploading the data as Supporting Information files, but we would recommend depositing data directly to a data repository if possible.

Please update your Data Availability statement in the submission form accordingly.

4. In the online submission form you indicate that your data is not available for proprietary reasons and have provided a contact point for accessing this data. Please note that your current contact point is a co-author on this manuscript. According to our Data Policy, the contact point must not be an author on the manuscript and must be an institutional contact, ideally not an individual. Please revise your data statement to a non-author institutional point of contact, such as a data access or ethics committee, and send this to us via return email. Please also include contact information for the third party organization, and please include the full citation of where the data can be found.

[Note: HTML markup is below. Please do not edit.]

Reviewers' comments:

Reviewer's Responses to Questions

**Comments to the Author**

1. Is the manuscript technically sound, and do the data support the conclusions?

The manuscript must describe a technically sound piece of scientific research with data that supports the conclusions. Experiments must have been conducted rigorously, with appropriate controls, replication, and sample sizes. The conclusions must be drawn appropriately based on the data presented. 

Reviewer #1: Partly

Reviewer #2: Yes

Reviewer #3: Yes

Reviewer #4: Yes

Reviewer #5: Partly

**********

2. Has the statistical analysis been performed appropriately and rigorously? 

Reviewer #1: Yes

Reviewer #2: I Don't Know

Reviewer #3: Yes

Reviewer #4: Yes

Reviewer #5: Yes

**********

3. Have the authors made all data underlying the findings in their manuscript fully available?

The PLOS Data policy requires authors to make all data underlying the findings described in their manuscript fully available without restriction, with rare exception (please refer to the Data Availability Statement in the manuscript PDF file). The data should be provided as part of the manuscript or its supporting information, or deposited to a public repository. For example, in addition to summary statistics, the data points behind means, medians and variance measures should be available. If there are restrictions on publicly sharing data—e.g. participant privacy or use of data from a third party—those must be specified.

Reviewer #1: No

Reviewer #2: No

Reviewer #3: No

Reviewer #4: No

Reviewer #5: No

**********

4. Is the manuscript presented in an intelligible fashion and written in standard English?

PLOS ONE does not copyedit accepted manuscripts, so the language in submitted articles must be clear, correct, and unambiguous. Any typographical or grammatical errors should be corrected at revision, so please note any specific errors here.

Reviewer #1: Yes

Reviewer #2: Yes

Reviewer #3: Yes

Reviewer #4: Yes

Reviewer #5: Yes

**********

5. Review Comments to the Author

Please use the space provided to explain your answers to the questions above. You may also include additional comments for the author, including concerns about dual publication, research ethics, or publication ethics. (Please upload your review as an attachment if it exceeds 20,000 characters)

Reviewer #1: In the abstract, it is mentioned that adults have been a little-studied population regarding the impact of COVID-19, but the study participants range in age from 18 to 78 years. To support the claim that this population is under-studied, I suggest specifying a more precise age range within this broad interval. Additionally, it would be helpful to detail which specific age groups were the most affected or how the sample was distributed by age, as this could help better contextualize the findings.

In the introduction, it is mentioned that COVID-19 has caused 3.78 million deaths to date, but it would be more accurate to cite the source that provides this figure, especially if referring to recent data up to 2025. This will help ensure the article aligns with the most up-to-date sources and prevent any potential confusion regarding the dates of the data.

In the introduction: The concept of Early Life Adversity (ELA) is mentioned, but I believe it could be characterized more clearly, particularly in terms of the different types of abuse (emotional, physical, sexual) and the specific characteristics of family dysfunction (e.g., domestic violence, parental separation or divorce, substance abuse, etc.). It would also be helpful to include the dimensions of the scales used to measure ELA, such as the ACE scale, to provide a more comprehensive overview of the methodological approach.

In the methodology: In the methods section, it is mentioned that the study worked with a population of adults aged 18 to 78, but this does not specifically clarify which age group is referred to when stating "under-studied." I would suggest including a more detailed breakdown by age groups to improve the understanding of the results.

Since online questionnaires were used, it is important to explain how barriers related to the participation of older adults or those with little experience in using electronic devices were overcome. Additionally, it would be relevant to address how the true voluntariness of the participants was ensured, considering whether the study adhered to the ethical principles of Helsinki. It would also be useful to explain how mobility restrictions due to the pandemic were addressed and how informed consent was obtained physically, particularly for the elderly population.

I believe it is important to address the issue of financial compensation for participants, especially in relation to the COVID-19 pandemic. It would be appropriate to explain how payment for completing the questionnaire was justified and whether this might have influenced participants' understanding or willingness to participate, particularly for those with less experience in virtual surveys.

For greater clarity and methodological rigor, I suggest including information about the validity and reliability of the scales used in the study. In particular, the ACE scale (Adverse Childhood Experiences) should mention its reliability alpha values (and, if applicable, McDonald's omega) to ensure the robustness of the measurements. The same should be done for the perceived threat of COVID-19 scale.

Since not all variables showed a normal distribution, it would be advisable to include a table with distribution statistics, such as skewness, kurtosis, and the results of the Kolmogorov-Smirnov normality test. This will help provide a clearer picture of the shape of the variable distributions and strengthen the interpretation of the statistical analyses.

Finally, since the study was conducted online, I suggest clarifying how informed consent was managed, especially with elderly participants. While consent was given electronically, it is important to ensure that the process was clearly explained to participants and that it was guaranteed they understood the process before giving their consent.

Reviewer #2: Which method or scale is used to assess the impact of the COVID-19 pandemic on this population? They only mention the use of the Adverse Childhood Experiences scale to assess two forms of early life adversity, abuse and family dysfunction.

Describe the topic and keywords.

This paragraph is very confusing to me "Participants’ state anxiety and coronavirus anxiety were

measured along with perceived threat from COVID-19.Additionally, as reduced

flexibility is implicated in the development and maintenance of anxiety disorders,

participants’ cognitive flexibility was assessed using the Cognitive Flexibility Inventory."

No indication of data collection methods and temporal space, as well as no reference to statistical analysis of the data.

Reviewer #3: This study explored the relationship between early life adversity (ELA) and state-level anxiety as well as coronavirus anxiety in a sample collected during the Covid-19 pandemic. Importantly, they tested whether the perceived threat from COVID-19 and perceived controllability during the pandemic has mediated the relationship between ELA and anxiety. Using parallel mediation analyses, the authors found that both perceived threat from COVID-19 and perceived controllability in the face of the pandemic mediated the relationship between ELA and adulthood state and coronavirus anxiety. I believe the results of this thoroughly conducted study is significant for the broader literature because 1) it replicates previous findings regarding the role of perceived threat in anxiety outcomes, and 2) more importantly it further expands our understanding of the role of controllability in mediating the relationship between ELA and anxiety outcomes.

This is a well-written and methodologically rigorous manuscript with clear and concise interpretations. It was a pleasure to read, and I believe it will be of significant value to both the broader audience and the scientific community. The study meaningfully advances our understanding of the relationship between early life experiences and anxiety in adulthood. That said, I believe several areas in the manuscript might be improved before publication. Below is a list of places where I thought clarifications are needed, as well as some recommendations for further discussion and improvement.

Introduction: The authors provide a strong overview of the relevant literature as it relates to their study design, however there are a few areas where the background could be expanded to better contextualize the study’s contributions.

• It would be helpful if the authors described state anxiety briefly in their introduction and discuss its role in relation to ELA before bringing up the Kalia et al study (lines 140-150). Given they also emphasize this characteristic in their own analyses, providing the relevant background on state anxiety as a construct would be beneficial for the general audience.

• Expanding the current study section to provide a more detailed description of the coronavirus anxiety and its relation to mental health problems would be beneficial. For instance, in line 157, please explain the functional impairments mentioned, examples of such impairments would give helpful context for the study.

• Further, to discuss their decision of parallel mediation models, a brief discussion of previous findings about the relationship between CFI-control and perceived threat (in general and particularly from COVID-19) is warranted. Why did the authors hypothesize that perceived controllability specifically would mediate the relationship between ELA and anxiety?

Methods:

• As a reviewer, I am curious whether the authors asked participants if they had lost any family members or close friends at the time of participation. While early life adversity (ELA) may, on its own, be expected to increase coronavirus-related anxiety, individuals low on the ELA scale who experienced the loss of loved ones due to COVID-19 may also exhibit elevated anxiety levels. This additional factor could contribute to the observed anxiety and may explain why a substantial portion of participants with a history of ELA reported maladaptive levels of coronavirus anxiety—potentially altering the interpretation of the findings. In other words, the experience of losing loved ones during the pandemic may account for additional variance in the relationship between ELA and coronavirus anxiety. If this variable was not assessed during data collection, I believe it still warrants acknowledgment as a limitation of the current study.

Analyses and results:

• The Methods section describes both the Alternatives and Control subscales of the CFI scale; however, the authors report results only from the CFI-Control subscale, without providing any rationale for excluding the CFI-Alternatives subscale from the analysis. It would be helpful to either explain the rationale for not analyzing the Alternatives subscale or to conduct and report additional models using the CFI-Alternatives subscale as a potential mediator, alongside the analyses involving the CFI-Control subscale.

• In the mediation analysis plan and results, the authors do not address whether the two mediators independently explain variance in the outcome. Specifically, did they assess the independence of the multiple-mediator assumption (i.e., low multicollinearity between mediators)? While it is appreciated that Table 1 reports the bivariate correlation between CFI-Control and perceived threat from COVID-19, and the observed correlation (r = .27) is modest and does not raise immediate concerns, it would be helpful for the authors to report variance inflation factor (VIF) or tolerance statistics to confirm that the mediators are not functionally redundant in predicting the outcome. Additionally, if any of the indirect effects reverse or disappear when mediators are modeled together, this may suggest overlapping variance and could impact interpretability; this possibility should be addressed in the discussion.

• Given that the manuscript reports results from four separate mediation models (with two independent and two dependent variables), each testing two mediators, I recommend that the authors address the potential for increased Type I error due to multiple comparisons. While each model may be theoretically justified and the authors appear cautious in interpreting their findings, reporting whether the results remain significant after correcting for multiple comparisons (e.g., Bonferroni or FDR adjustment) would enhance the transparency and interpretability of the findings.

• Finally, please make sure the beta coefficients reported in the parallel mediation models (in text) match the values reported in the corresponding figures (e.g., there is a slight mismatch between total and direct effect values reported in section 3.3.2 and Fig 6).

Reviewer #4: I am thankful for the opportunity to review this manuscript examining the relationship between adverse childhood experiences and coronavirus anxiety in a large sample of adults. Overall, I think the manuscript is well written and easy to follow, with a strong background and clear aims. It will make a nice contribution to the existing literature. I just have a few minor comments for the authors to consider.

1. The authors hypothesize that ELA, in the form of both maltreatment and household dysfunction, would predict higher state and coronavirus anxiety. However, this study is based on a previous published paper that found such a relationship (in the context of COVID-19 threat) for maltreatment only, not household dysfunction. Why then, was household dysfunction predicted to have an impact?

2. Were power analyses conducted?

3. In the methods, some example questionnaire items are italicized, while others are not. Please make consistent. The same can be said for the Likert scale anchors; please also make consistent.

4. Is it possible to calculate a Cronbach’s alpha for the perceived threat of COVID-19, since the three items were summed?

5. The methods and data analysis sections switch back and forth between past, present, and future tense. Please make consistent.

6. I think there is a typo on line 346. The authors write, “When mediators were included in the model, the direct effect of maltreatment on state anxiety remained significant”. However, this is the section talking about household dysfunction, so I believe the term maltreatment should be changed household dysfunction here? Looking further down, I see the same exact typo on line 366. Please correct.

Reviewer #5: Thank you for the opportunity to review this manuscript. The topic addressed is highly relevant and timely, particularly in the context of understanding the long-term psychological effects of early life adversity and the role of cognitive flexibility in responding to environmental stressors such as the COVID-19 pandemic. However, I have several serious concerns regarding the manuscript’s novelty, transparency, and research ethics that should be carefully considered before further evaluation. These are outlined below:

Major concerns:

1. Claimed replication and extension is misleading

The authors state that they aimed to "replicate the report by Kalia and colleagues [7]" and "extend the work [...] by examining the relation between ELA and coronavirus anxiety." (lines 152-155). However, Kalia et al. (2020) [7] already examined the relation between early life adversity, perceived threat of COVID-19, and state anxiety using the same variables and measures—including items assessing perceived threat and self-report anxiety. The notion that coronavirus anxiety is a new addition is not supported by the evidence, as the original study operationalized threat appraisal through direct questions about COVID-19 severity, impact, and personal risk—items that are reused verbatim here. This undermines the claim of novel contribution.

2. Undisclosed authorship overlap with prior study

The authors do not clearly disclose that they are the same researchers who conducted the earlier study they claim to replicate. This omission is ethically problematic, especially in the context of research transparency. If the present manuscript is based on the same or overlapping team and intellectual framework as [7], this should be clearly stated in the manuscript.

3. Single IRB protocol number

The study presented here was approved under the same Institutional Review Board (IRB) protocol as the study published in 2020 [7]. If this is the case, the current study cannot be considered a replication in the conventional sense, as it was not independently planned and conducted after the original study's publication. Rather, it appears to be a second analysis (or a re-analysis) under the same ethical approval. This further undermines the framing of this work as a replication and extension.

4. Lack of data availability and transparency

The dataset is not made publicly available, and no justification is provided for this decision. According to APA guidelines (Publication Manual, 7th ed., §1.14; JARS) and PLOS ONE’s own data availability policies, authors are expected to retain and share their data unless there are explicit ethical, legal, or contractual constraints—constraints which must be clearly described in the manuscript. In this case, the data consist of anonymizable self-report survey responses, and thus appear eligible for responsible sharing. If the restriction is due to an IRB decision, I request that the justification for such a restriction be included, as withholding the data without a clear ethical rationale contradicts principles of scientific transparency and reproducibility.

5. Substantial conceptual and structural overlap with previously published article

The Introduction section in the current manuscript closely mirrors the structure, content, and key arguments of the Introduction from the authors’ earlier publication [7]. While the wording has been paraphrased and references partially updated, the conceptual framework, logical flow, and supporting literature remain largely the same. Although self-citation and continuity of ideas are common in programmatic research, the extent of overlap here raises concerns about the manuscript’s originality. At minimum, the manuscript should more clearly acknowledge the derivative nature of its framing and literature background, and the Introduction should be revised to ensure the presentation of a novel and independent rationale.

6. Misleading use of mortality statistics and incorrect citation of source.

The opening sentence of the Introduction states that “The COVID-19 pandemic has been unprecedented, with 3.78 million deaths worldwide (600,000 deaths in the US) to date,” citing Twenge and Joiner (2020) [1]. However, this reference does not report any COVID-19 death statistics. Furthermore, it was published in the early stages of the pandemic and could not have contained accurate mortality data of that magnitude. The use of “to date” misleadingly implies that the figures are current as of 2025, which they are not. This misrepresents the cited source and may compromise the manuscript’s credibility. If such statistics are to be included, they should be based on official, up-to-date sources (e.g., WHO, CDC) and cited appropriately.

**********

6. PLOS authors have the option to publish the peer review history of their article (what does this mean?). If published, this will include your full peer review and any attached files.

If you choose “no”, your identity will remain anonymous but your review may still be made public.

**Do you want your identity to be public for this peer review?** For information about this choice, including consent withdrawal, please see our Privacy Policy.

Reviewer #1: No

Reviewer #2: **Yes: **Ana Carina Henriques Teodósio Moisão

Reviewer #3: No

Reviewer #4: No

Reviewer #5: **Yes: **Andrzej Śliwerski

**********

[NOTE: If reviewer comments were submitted as an attachment file, they will be attached to this email and accessible via the submission site. Please log into your account, locate the manuscript record, and check for the action link "View Attachments". If this link does not appear, there are no attachment files.]

While revising your submission, please upload your figure files to the Preflight Analysis and Conversion Engine (PACE) digital diagnostic tool, https://pacev2.apexcovantage.com/. PACE helps ensure that figures meet PLOS requirements. To use PACE, you must first register as a user. Registration is free. Then, login and navigate to the UPLOAD tab, where you will find detailed instructions on how to use the tool. If you encounter any issues or have any questions when using PACE, please email PLOS at figures@plos.org. Please note that Supporting Information files do not need this step.

# Author comment: Author response to Decision Letter 1

**DOI:** 10.1371/journal.pone.0323401.r003

4 Sep 2025

See attached letter.

# Peer review: Decision Letter 1

**DOI:** 10.1371/journal.pone.0323401.r004

**Authors:** Rosemary Bassey

5 Oct 2025

Adults with Adverse Childhood Experiences Report Greater Coronavirus Anxiety

PONE-D-25-13890R1

Dear Dr. Vrinda Kalia,

We’re pleased to inform you that your manuscript has been judged scientifically suitable for publication and will be formally accepted for publication once it meets all outstanding technical requirements.

Within one week, you’ll receive an e-mail detailing the required amendments. When these have been addressed, you’ll receive a formal acceptance letter and your manuscript will be scheduled for publication.

An invoice will be generated when your article is formally accepted. Please note, if your institution has a publishing partnership with PLOS and your article meets the relevant criteria, all or part of your publication costs will be covered. Please make sure your user information is up-to-date by logging into Editorial Manager at Editorial Manager® and clicking the ‘Update My Information' link at the top of the page. For questions related to billing, please contact billing support.

If your institution or institutions have a press office, please notify them about your upcoming paper to help maximize its impact. If they’ll be preparing press materials, please inform our press team as soon as possible -- no later than 48 hours after receiving the formal acceptance. Your manuscript will remain under strict press embargo until 2 pm Eastern Time on the date of publication. For more information, please contact onepress@plos.org.

Kind regards,

Rosemary Bassey, Ph.D.

Academic Editor

PLOS ONE

Additional Editor Comments (optional):

Reviewers' comments:

Reviewer's Responses to Questions

**Comments to the Author**

1. If the authors have adequately addressed your comments raised in a previous round of review and you feel that this manuscript is now acceptable for publication, you may indicate that here to bypass the “Comments to the Author” section, enter your conflict of interest statement in the “Confidential to Editor” section, and submit your "Accept" recommendation.

Reviewer #1: All comments have been addressed

Reviewer #3: All comments have been addressed

Reviewer #4: All comments have been addressed

**********

2. Is the manuscript technically sound, and do the data support the conclusions?

The manuscript must describe a technically sound piece of scientific research with data that supports the conclusions. Experiments must have been conducted rigorously, with appropriate controls, replication, and sample sizes. The conclusions must be drawn appropriately based on the data presented. 

Reviewer #1: Yes

Reviewer #3: Yes

Reviewer #4: Yes

**********

3. Has the statistical analysis been performed appropriately and rigorously? 

Reviewer #1: Yes

Reviewer #3: Yes

Reviewer #4: Yes

**********

4. Have the authors made all data underlying the findings in their manuscript fully available?

The PLOS Data policy requires authors to make all data underlying the findings described in their manuscript fully available without restriction, with rare exception (please refer to the Data Availability Statement in the manuscript PDF file). The data should be provided as part of the manuscript or its supporting information, or deposited to a public repository. For example, in addition to summary statistics, the data points behind means, medians and variance measures should be available. If there are restrictions on publicly sharing data—e.g. participant privacy or use of data from a third party—those must be specified.

Reviewer #1: Yes

Reviewer #3: No

Reviewer #4: No

**********

5. Is the manuscript presented in an intelligible fashion and written in standard English?

PLOS ONE does not copyedit accepted manuscripts, so the language in submitted articles must be clear, correct, and unambiguous. Any typographical or grammatical errors should be corrected at revision, so please note any specific errors here.

Reviewer #1: Yes

Reviewer #3: Yes

Reviewer #4: Yes

**********

6. Review Comments to the Author

Please use the space provided to explain your answers to the questions above. You may also include additional comments for the author, including concerns about dual publication, research ethics, or publication ethics. (Please upload your review as an attachment if it exceeds 20,000 characters)

Reviewer #1: Great job to the authors on addressing all the comments. Congratulations on the new publication as well

Reviewer #3: The authors have thoroughly addressed my previous questions and concerns. I find the revised manuscript to be substantially improved and believe it is ready for publication.

Reviewer #4: I thank the authors for their thorough revisions. I am satisfied with the revisions and have no further comments.

**********

7. PLOS authors have the option to publish the peer review history of their article (what does this mean?). If published, this will include your full peer review and any attached files.

If you choose “no”, your identity will remain anonymous but your review may still be made public.

**Do you want your identity to be public for this peer review?** For information about this choice, including consent withdrawal, please see our Privacy Policy.

Reviewer #1: **Yes: **Wilson Guillermo Siguenza Campoverde

Reviewer #3: No

Reviewer #4: No

**********

# Editor report: Acceptance letter

**DOI:** 10.1371/journal.pone.0323401.r005

**Authors:** Rosemary Bassey

PONE-D-25-13890R1

PLOS ONE

Dear Dr. Kalia,

I'm pleased to inform you that your manuscript has been deemed suitable for publication in PLOS ONE. Congratulations! Your manuscript is now being handed over to our production team.

At this stage, our production department will prepare your paper for publication. This includes ensuring the following:

* All references, tables, and figures are properly cited

* All relevant supporting information is included in the manuscript submission,

* There are no issues that prevent the paper from being properly typeset

You will receive further instructions from the production team, including instructions on how to review your proof when it is ready. Please keep in mind that we are working through a large volume of accepted articles, so please give us a few days to review your paper and let you know the next and final steps.

Lastly, if your institution or institutions have a press office, please let them know about your upcoming paper now to help maximize its impact. If they'll be preparing press materials, please inform our press team within the next 48 hours. Your manuscript will remain under strict press embargo until 2 pm Eastern Time on the date of publication. For more information, please contact onepress@plos.org.

You will receive an invoice from PLOS for your publication fee after your manuscript has reached the completed accept phase. If you receive an email requesting payment before acceptance or for any other service, this may be a phishing scheme. Learn how to identify phishing emails and protect your accounts at https://explore.plos.org/phishing.

If we can help with anything else, please email us at customercare@plos.org.

Thank you for submitting your work to PLOS ONE and supporting open access.

Kind regards,

PLOS ONE Editorial Office Staff

on behalf of

Dr. Rosemary Bassey

Academic Editor

PLOS ONE
